# Frailty as a risk marker of adverse lower urinary symptom outcomes in patients with benign prostatic hyperplasia undergoing transurethral resection of prostate

**DOI:** 10.3389/fmed.2023.1185539

**Published:** 2023-05-19

**Authors:** Xiang Ren, Jing Wang, Zhixian Wang, Yisheng Yin, Xing Li, Yiqun Tian, Zihao Guo, Xiaoyong Zeng

**Affiliations:** ^1^Department of Urology, Tongji Hospital, Tongji Medical College, Huazhong University of Science and Technology, Wuhan, China; ^2^Department of Urology, Wuhan Hospital of Traditional Chinese and Western Medicine, Tongji Medical College, Huazhong University of Science and Technology, Wuhan, China; ^3^Department of Urology, Wuhan No. 1 Hospital, Wuhan, China

**Keywords:** frailty, benign prostatic hyperplasia, transurethral resection of prostate, lower urinary tract symptoms, propensity score matching

## Abstract

**Purpose:**

Lower urinary symptoms (LUTS) may persist in a proportion of patients with benign prostatic hyperplasia (BPH) following transurethral resection of prostate (TURP), which is a major cause of reduced quality-of-life. We aimed to investigate the effect of frailty on LUTS in patients with BPH treated with TURP.

**Methods:**

We longitudinally evaluated LUTS and health-related quality-of-life (HRQOL) in patients with BPH treated with TURP from February 2019 and January 2022 using International Prostate Symptom Score (IPSS) and Short Form-8 (SF-8), respectively. Patients were divided into frail and non-frail groups according to the Fried phenotype (FP). The primary purpose was comparing the outcomes of LUTS and HRQOL between two groups. Secondary purposes were investigating the frailty as a preoperative predictor of postoperative adverse LUTS outcomes following TURP using logistic regression analysis. A 1:2 propensity score matching (PSM) was performed to reduce the effects of selection bias and potential confounders.

**Results:**

Of the 567 patients enrolled, 495 (87.3%) patients were non-frail (FP = 0–2), and the remaining 72 (12.7%) patients were classified into the frail group. There were no significant differences in body mass index (BMI), urine white blood cell (UWBC), creatinine, prostate-specific antigen (PSA) and prostate volume in both groups at baseline (all *p* > 0.05). However, patients with frailty were older, higher comorbidity rates, lower peak flow rates and lower HRQOL. In the frail group, although LUTS and HRQOL at 6 months following TURP improved significantly compared to those at baseline, it did not show a significant improvement compared with the non-frail group (both *p* < 0.001). Moreover, multivariable logistic regression analysis demonstrated that preoperative frailty was significantly associated with poor LUTS improvement in both the entire cohort and PSM subset (both *p* < 0.05), whereas age and comorbidities were not after PSM analysis.

**Conclusion:**

In patients with frail or non-frail, TURP for BPH provides overall good results. However, frail individuals are at higher risk of postoperative adverse LUTS outcomes. Frailty has the potential to be a strong objective tool for risk stratification and should be considered during the perioperative evaluation.

## Introduction

Benign prostatic hyperplasia (BPH) is a leading cause of male lower urinary tract symptoms (LUTS), which severely affects the quality of life of middle-aged and elderly men (1, 2). It has been reported that BPH occurs in 15 to 60% of men over the age of 40, and that the prevalence of BPH increases markedly with age (3, 4). Consequently, the burden of BPH on the global healthcare system will remain to grow over the next few decades, and thus increased management will be necessary to treat the condition.

Surgical intervention for BPH used to be a consideration for patients who have failed to respond to medical management or for those who have complications from obstruction of the bladder outlet due to BPH (5). Although transurethral resection of prostate (TURP), the gold standard for the surgical treatment of BPH, remain significant symptom improvement (6, 7), 20–50% of patients still experience persistent LUTS after TURP (8, 9). There have been a study exploring potential risk factors for re intervention in the treatment of BPH with TURP for long term follow-up (10). In addition, because of the importance for the surgeon and patients to know which group of patients is at risk for poor outcomes after TURP, numbers of studies have also evaluated predictors that may cause decline in the surgical success rate (8, 11–13). However, the majority of these previous studies have limited to focus on the association between superficial demographic characteristics and postoperative complications (14), the data related to LUTS improvement is still scarce and there is a critical need for diagnostic tests that can distinguish LUTS outcomes from a systemic cause (15).

Recently, interest in the association between frailty and urological diseases has been increasing since elevated numbers of elderly patients worldwide (16–19). Frailty, which can be defined as a biological syndrome of diminished reserves and reduced resistance to stressors, caused by cumulative declines in multiple physiological systems and resulting in vulnerability to adverse outcomes (20). In addition, previous literature has suggested that frailty is a systemic marker of biological age, potentially mediating the well-established association between chronological age and LUTS (15). However, frailty is not yet targeted by any existing male LUTS interventions (21, 22), and thus frailty might represent a promising novel therapeutic predictor that should be further evaluated (15).

With this in mind, we investigated the LUTS and health-related quality-of-life (HRQOL) outcomes of TURP in patients with preoperative frailty or not frailty. Actually, our results confirmed that frailty was a strong predictor for poor postoperative LUTS improvement in BPH patients using propensity score matching (PSM) analysis. This determination might be helpful in the management of expectations of patient and surgeon after TURP.

## Materials and methods

### Study population

The retrospective longitudinal study was conducted on 567 of the 654 recruitable patients between February 2019 and January 2022 from the Department of Urology, Huazhong University of Science and Technology Tongji Hospital. The patients who used regularly α-receptor blockers and 5α-reductase inhibitors but without a satisfactory LUTS improvement or patients who do not tolerate drug therapy were enrolled in the study. Meanwhile, the inclusion criteria also required patients with BPH who fulfilled frailty screening, IPSS and HRQOL questionnaires at baseline and 6 months after TURP. All of the patients enrolled in the study underwent a standard TURP procedure performed by the experienced surgical team of our department. The exclusion criteria were as follows: (1) patients with a neurogenic bladder, (2) patients with an unstable bladder, (3) patients once undergoing TURP, (4) patients with detrusor weakness, (5) patients with an anterior urethral stricture, and (6) patients with preoperative urethral external sphincter injury. This study was conducted in accordance with the Declaration of Helsinki and was approved by Ethics Committee of Tongji Hospital. Informed consent was obtained from all participants.

### Variables collection and definition

Demographic characteristics, clinical features and laboratory data, including age, BMI, preoperative comorbidity, peak uroflowmetry data (Qmax), PSA, urine white blood cell (WBC), creatinine, prostate volume, IPSS, and HRQOL score, were retrieved within 24 h after hospitalization and 6 months after the surgery. We assessed frailty using the Fried phenotype (FP) (20), which includes five items: weight loss, low physical activity, slow gait speed, handgrip weakness and exhaustion. The FP ranges from 0 to 5, and FP ≥ 3 was defined as frail (20). The preoperative comorbidity was defined according to the Charlson comorbidity index (CCI) (23) and was categorized as CCI 0–1 vs. CCI ≥ 2. The IPSS is a questionnaire widely used to screen, diagnose and monitor symptoms of benign prostatic hyperplasia, which was used for LUTS evaluation (24). Here, the IPSS was classified into mild (0–7 points), moderate (8–19 points), and severe (20–35 points) categories, and the poor improvement of LUTS after surgery was defined as a <50% decrease in total IPSS of at follow-up compared with baseline (11, 25). The Short Form-8 (SF-8), including physical component summary (PCS) and the mental component summary (MCS), is seen as a standard instrument that is easy to clinically determine health status quality-of-life within a short period of time, as used for assessment of HRQOL score in the study (26), and the higher scores indicate better status.

### PSM analysis

Since patients in the current study were allocated on the basis of their fried phenotype rather than randomly, selection bias and potential confounders would reduce the reliability of the results. Therefore, PSM was used for random assignment simulation and minimization of effects. Propensity scores were estimated on the basis of a logistic regression model according to the baseline characteristics, including age, CCI, Qmax, IPSS, and SF-8. A 0.2 caliper was used for one-to-two matching.

### Outcomes

The present study primarily aimed to compare the difference in IPSS and HRQOL before and 6 months after TURP between the frail and nonfrail groups. Our secondary purpose was to investigate prognostic impact of frailty on LUTS improvement in BPH following TURP.

### Statistical analysis

Data were demonstrated as mean ± standard deviation for continuous variables and proportions for categorical variables. Data were compared using a paired *t*-test or the Wilcoxon signed rank test for groups not normally distributed. Comparation of categorical variables were performed using the Fisher’s exact test or the *χ*^2^ test. Multivariate analysis performed by logistic regression analysis was used to identify the independent parameters of poor LUTS results after surgery. The odds ratio (OR) and 95% confidence interval (CI) were calculated. All statistical analyses were carried out by using SPSS 22.0. *p* < 0.05 was considered statistically significant.

## Results

### Patient selection and baseline characteristics of the participants

**Figure 1:**
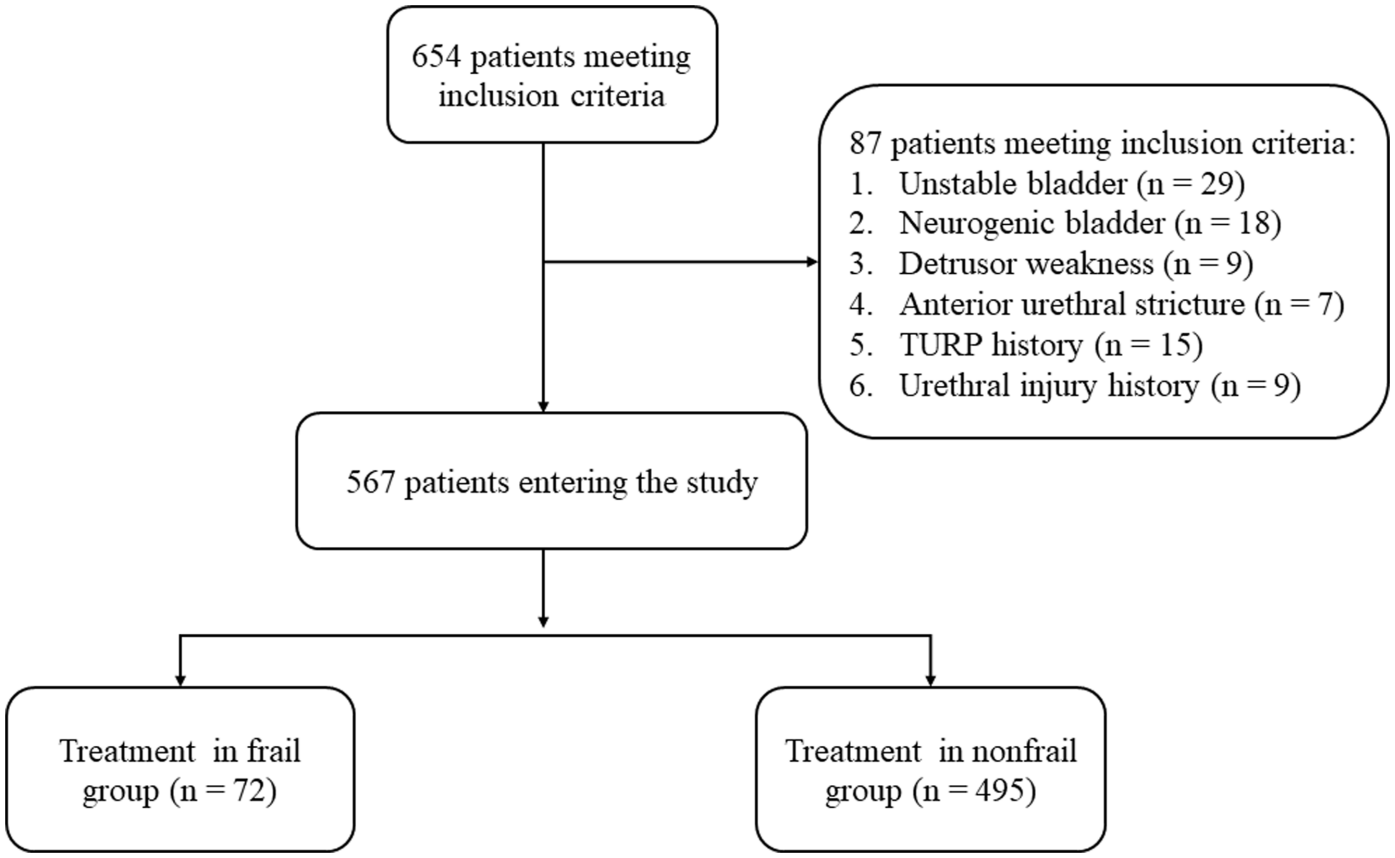
Flow chart.

Over the 2 year follow-up period of the study, a total of 654 patients met the inclusion criteria. Among them, 87 were excluded owing to the presence of unstable bladder (*n* = 29), neurogenic bladder (*n* = 18), detrusor weakness (*n* = 9) and anterior urethral stricture (*n* = 7). In addition, we excluded 24 patients who had experienced TURP surgery (*n* = 15) and preoperative urethral external sphincter injury (*n* = 9). Finally, the remaining 567 patients were divided into the frail (*n* = 72) and nonfrail (*n* = 495) groups (Figure 1).

Of the 567 patients, the median age of the frail group was significantly higher than that of the nonfrail group (75.2 vs. 72.5 years, *p* = 0.035). Moreover, the rates of comorbidities, such as hypertension (53.1% vs. 50.2%), respiratory diseases (20.6% vs. 8.9%), tumors (excluding prostate and bladder tumors) (16.7% vs. 7.3%), diabetes mellitus and cardiovascular disease (32.2% vs. 28.1%), were significantly higher in the frail group than in the non-frail group, namely, with a summative higher CCI (*p* = 0.005). The frail group showed a higher proportion of patients with severe LUTS than the nonfrail group (44.4% vs. 39.0%), while the general IPSS were not significantly different between the groups (*p* = 0.356). In addition, the frail group was associated with a lower preoperative peak flow rate, PCS, and MCS (all *p* < 0.05). The BMI, WBC, creatinine, PSA and prostate volume were not significantly different between the frail and nonfrail group (all *p* > 0.05) (Table 1).

**Table 1:** Baseline characteristics of the participants.

Variables	Frail (*n* = 72)	Nonfrail (*n* = 495)	*p* value
Age (years)	75.2 ± 8.9	72.5 ± 10.3	0.035
BMI (kg/m^2^)	25.5 ± 5.5	26.3 ± 6.1	0.293
WBC (10^9^/L)	8.4 ± 4.3	7.6 ± 5.1	0.206
Creatinine (μmol/L)	75.7 ± 10.3	73.6 ± 8.8	0.065
PSA, ng/mL	2.6 ± 1.3	2.5 ± 1.5	0.592
CCI, *n* (%)			0.005
0–1	16 (22.2)	194 (39.2)	
≥2	56 (77.8)	301 (60.8)	
Prostate volume (mL)	67.4 ± 15.5	68.4 ± 14.3	0.584
Qmax (mL/s)	5.8 ± 3.2	7.1 ± 3.6	0.004
IPSS, *n* (%)			0.356
Mild	18(25.0)	144(29.1)	
Moderate	22(30.6)	158(31.9)	
severe	32(44.4)	193(39.0)	
FP	4.2 ± 0.9	2.0 ± 0.8	<0.001
SF-8			
PCS	28.9 ± 11.5	37.3 ± 12.9	<0.001
MCS	33.3 ± 10.8	37.7 ± 9.5	<0.001

BMI, body mass index; WBC, white blood cell; PSA, prostate-specific antigen; CCI, Charlson comorbidity index; Qmax, peak flow rate; IPSS, International Prostate Symptom Score; FP, fried phenotype; SF-8, Short Form-8; PCS, physical component summary; MCS, mental component summary.

### Perioperative LUTS and HRQOL outcomes stratified by frailty

Frail patients and non-frail patients both exhibited significant differences in the IPSS between the baseline and 6 months follow-up (both *p* < 0.001). However, a significant difference was observed between the frail and non-frail groups at 6 months following TURP (17.3 ± 5.5 vs. 6.8 ± 3.6, *p* < 0.001). Similarly, there was a statistical evidence in the HRQOL between the baseline and 6 months follow-up in both frail and non-frail groups. Also, at 6 months following TURP, HRQOL was higher in non-frail patients compared with frail patients (87.5 ± 12.5 vs. 67.3 ± 14.5, *p* < 0.001). Moreover, in order to reflect the urination of patients more objectively, we additionally compared detailed measurement of peak flow rate in the two groups. Finally, the results also showed that postoperative Qmax compared to baseline was significantly different (*p* < 0.001), while Qmax was lower in the frailty patients compared to non-frail patients (4.4 ± 4.9 vs. 10.4 ± 4.1, *p* < 0.001) (Table 2).

**Table 2:** Follow-up characteristics of LUTS, HRQOL, and Qmax in both frail and nonfrail groups.

	Frail (*n* = 72)	Nonfrail (*n* = 495)	*p* value
IPSS			
Preoperative	27.1 ± 6.9	25.4 ± 7.5	0.070
Postoperative (6 mo)	17.3 ± 5.5	6.8 ± 3.6	<0.001
*p* value	<0.001	<0.001	
Difference in IPSS	9.7 ± 5.8	18.6 ± 5.1	<0.001
HRQOL			
Preoperative	62.2 ± 11.0	75.1 ± 11.3	<0.001
Postoperative (6 mo)	67.3 ± 14.5	87.5 ± 12.5	<0.001
*p* value	0.019	<0.001	
Difference in HRQOL	5.1 ± 9.3	12.4 ± 11.9	<0.001
Qmax (mL/s)			
Preoperative	5.8 ± 3.2	7.1 ± 3.6	0.004
Postoperative (6 mo)	10.2 ± 5.1	17.6 ± 4.5	<0.001
*p* value	<0.001	<0.001	
Difference in Qmax	4.4 ± 4.9	10.4 ± 4.1	<0.001

LUTS, lower urinary tract symptoms; HRQOL, health-related quality-of-life; IPSS, International Prostate Symptom Score; Qmax, peak flow rate.

### Independent prognostication for poor improvement of LUTS

We performed univariate and multivariate analyses for the prediction of poor improvement of LUTS in the study as given in Table 3 unmatched group. After examining the univariate significant difference indicators, age (*p* = 0.009), CCI (*p* = 0.031), prostate volume (*p* < 0.001), Qmax (*p* < 0.001), IPSS (*p* = 0.005), FP (*p* < 0.001), PCS (*p* = 0.014), and MCS (*p* < 0.001) in the SF-8 were fit to a multiple logistic regression analysis, which showed that frailty was significantly associated with poor improvement of LUTS (OR = 1.63 [1.10–2.98]; *p* < 0.001). In addition, age (OR = 1.35 [1.10–1.89]; *p* = 0.025), Qmax (OR = 0.59 [0.49–0.88]; *p* = 0.002), IPSS (OR = 1.21 [1.07–1.54]; *p* = 0.038), and MCS (OR = 0.68 [0.54–1.42]; *p* = 0.001) were independent predictors of poor results. Other parameters, such as CCI, prostate volume and PCS were not found to be of independent significance.

**Table 3:** Univariate and multivariate analysis for poor improvement of LUTS in BPH patients after TURP.

Variables	OR (95% CI)	Univariate analysis *p*	OR (95% CI)	Multivariate analysis *p*
Unmatched group (*n* = 567)				
Age	1.46 (1.16–2.15)	0.009	1.35 (1.10–1.89)	0.025
BMI	6.01 (3.90–9.20)	0.256		
WBC	3.79 (2.35–6.49)	0.566		
Creatinine	1.54 (1.21–2.78)	0.545		
PSA	1.65 (1.49–2.00)	0.079		
CCI (≥2 vs. 0–1)	1.06 (0.95–1.26)	0.031	0.95 (0.88–1.17)	0.046
Prostate volume	1.83 (1.21–3.57)	<0.001	1.59 (1.35–4.15)	0.063
Qmax	0.63 (0.52–0.76)	<0.001	0.59 (0.49–0.88)	0.002
IPSS	1.37 (1.13–1.84)	0.005	1.21 (1.07–1.54)	0.038
FP (frail vs. nonfrail)	2.12 (1.19–3.55)	<0.001	1.63 (1.10–2.98)	<0.001
PCS	0.90 (0.43–1.52)	0.014	0.79 (0.40–1.49)	0.069
MCS	0.77 (0.55–1.48)	<0.001	0.68 (0.54–1.42)	0.001
Matched group (*n* = 153)				
Age	1.32 (1.04–1.95)	0.015	1.23 (0.98–1.93)	0.233
BMI	2.33 (1.76–4.33)	0.435		
WBC	2.87 (1.35–4.55)	0.611		
Creatinine	1.76 (1.11–2.48)	0.577		
PSA	0.93 (0.86–2.01)	0.153		
CCI (≥2 vs. 0–1)	1.17 (1.01–1.26)	0.054		
Prostate volume	1.69 (1.15–3.83)	0.009	1.44 (1.06–2.56)	0.349
Qmax	0.55 (0.52–1.26)	<0.001	0.43 (0.35–1.17)	0.061
IPSS	1.39 (1.21–1.97)	0.056		
FP (frail vs. nonfrail)	3.5 (1.47–4.23)	<0.001	1.49 (1.06–2.79)	0.026
PCS	1.23 (0.87–1.94)	0.197		
MCS	0.69 (0.48–2.18)	0.028	0.54 (0.49–1.32)	0.069

OR, odds ratio; CI, confidence interval; BMI, body mass index; WBC, white blood cell; PSA, prostate-specific antigen; CCI, Charlson comorbidity index; Qmax, peak flow rate; IPSS, International Prostate Symptom Score; FP, fried phenotype; SF-8, Short Form-8; PCS, physical component summary; MCS, mental component summary.

### The prognostic significance of frailty after PSM

Because of the imbalance in a number of variables between frail and nonfrail patients, we performed a 1:2 ratio PSM to reduce potential confounding. In the PSM analysis, 51 patients from frail group were matched pairs with 102 patients from nonfrail using the nearest-neighbor algorithm. The clinical characteristics and laboratory parameters of the two groups of patients were well balanced and evenly distributed (all *p* > 0.1, Table 4). Finally, the multivariate analyses showed that frailty still remained an independent predictor of poor improvement of LUTS (OR = 1.49 (1.06–2.79); *p* = 0.026), whereas age, Qmax, comorbidities, preoperative IPSS and HRQOL were not (all *p* > 0.05, Table 3 matched group).

**Table 4:** Baseline characteristics in the PSM cohort.

Variables	Frail (*n* = 51)	Nonfrail (*n* = 102)	*p* value
Age (years)	74.5 ± 8.8	73.6 ± 9.5	0.572
BMI (kg/m^2^)	26.0 ± 5.4	26.7 ± 6.2	0.494
WBC (10^9^/L)	8.2 ± 4.2	7.6 ± 5.0	0.463
Creatinine (μmol/L)	75.3 ± 9.5	73.6 ± 9.1	0.285
PSA, ng/mL	2.6 ± 1.5	2.5 ± 1.6	0.710
CCI, *n* (%)			0.489
0–1	19 (37.3)	44 (43.1)	
≥2	32 (62.7)	58 (56.9)	
Prostate volume (mL)	67.5 ± 16.1	68.3 ± 13.6	0.748
Qmax (mL/s)	6.1 ± 3.4	7.3 ± 3.9	0.068
IPSS, *n* (%)			0.086
Mild	14(27.4)	36(35.3)	
Moderate	16(31.4)	40(39.2)	
Severe	21(41.2)	26(25.5)	
FP	3.9 ± 1.5	2.2 ± 1.3	<0.001
SF-8			
PCS	30.9 ± 10.5	34.6 ± 12.6	0.073
MCS	34.7 ± 11.0	35.9 ± 10.1	0.502

BMI, body mass index; WBC, white blood cell; PSA, prostate-specific antigen; CCI, Charlson comorbidity index; Qmax, peak flow rate; IPSS, International Prostate Symptom Score; FP, fried phenotype; SF-8, Short Form-8; PCS, physical component summary; MCS, mental component summary.

## Discussion

As the patient with BPH continues to increase due to aging, it is increasingly important to identify increased risk stratification in the perioperative period. Accumulating evidence has implicated that frailty can increase vulnerability to treatment-related adverse outcomes. However, the effect of frailty on postoperative LUTS improvement in patients with BPH is not well established. In the present study, we discovered that frailty was significantly associated with LUTS and HRQOL in 6 months following TURP, and frailty was an independent predictor of poor improvement of LUTS after surgery, which indicated the importance of considering frailty in the management of common urologic symptoms.

Since frailty is the result of a cumulative decline across multiple organ systems, often leading to deterioration and adverse events when responsing to stressors such as surgery (27), in recent years frailty has been used as a screening tool to predict the outcomes after major surgery. However, there still has been lack of a standardized and valid method to screen those who are truly frail so that they can be effectively targeted for identification and care. Due to the fact that patients with BPH are usually older, in this study, we used a standardized, physiologically-based success definition of frailty that fits the spectrum of frailty manifestations seen in older adults and that usually be used to establish clinical risk of adverse outcomes (20). Frailty screening can improve prediction of individuals undergoing general surgical (28–30) and urological procedures (18, 31–33) at high risk for poor surgical outcomes. Recently, one study performed in the Aging Study of PyeongChang Rural Area found older men with LUTS had a higher prevalence of frailty and geriatric conditions, however, postoperative outcomes were not evaluated separately in this study (34). Intriguingly, some prior studies examined patients undergoing elective surgical procedures and found that preoperative frailty was associated with an increased risk of postoperative complications, length of hospital stay, and 30 day morbidity and mortality (35). Accordingly, our findings are similarly consistent with these studies examining the association between frailty and poorer postoperative outcomes.

Although evidence on the effect of frailty on postoperative LUTS and HRQOL in patients with urological procedures is lacking, a recent study showed that frailty was not significantly associated with the worsening of LUTS and HRQOL in prostate cancer patients undergoing robot-assisted radical prostatectomy (RARP) by multivariable logistic regression analysis, whereas in the frailty group, LUTS at 12 months following RARP did not significantly improve compared to those at the baseline (32).We think it may be the reason that LUTS occur only when the tumor obstructs the urethra or invades the bladder neck, and it lack significant associations with the risk of prostate cancer (36, 37). An additional reason may be that patients with frailty may be related to the indication for surgery, and the impact of frailty on postoperative outcomes may also differ by type of surgery (38, 39), emphasizing the importance of assessing frailty among all patients undergoing any type of urologic surgery. Anyway, the results also showed that patients with frail had a worse postoperative LUTS and HRQOL recovery than the nonfrail patients.

Although many definitions of successful TURP outcome exist in the literature, most papers define it as an improvement from baseline on the International Prostate Symptom Score (IPSS) (40, 41). Recently, an Italian cohort study used a benign prostatic obstruction nomogram to well predict the postoperative outcome of TURP according to IPSS (42). In our study, IPSS in both frail patients and non-frail patients recovered to different degrees during the follow-up period, but the non-frail group improved more significantly. We think the reason may be that one of the factors in assessing PF is muscle weakness, such as grip strength, low physical activity and walking slowness (20), so LUTS outcome of frail old people may be mainly caused by sarcopenia (17). As a key component of frailty, sarcopenia increases the risk of LUTS (43). Also, the slowed gait and falling experience of were important causes of PF, and these can be attributed to progressive loss of both motor nerves and muscle fibres as well as impaired myocyte function with age (44). Besides, urination is controlled by central nervous system-affected sacral nerve urinary reflexes (43), and LUTS is considered to be a frequent complaint in old males with a major impact on HRQOL (45). Therefore, nervous system dysfunction might be another significant cause. Collectively, these findings extend and corroborate the present study on the highly predictive of frailty to postoperative LUTS and HRQOL outcomes.

Additionally, increasing age is one of the potential LUTS risk factors, and older men with severe urologic symptoms were more likely to be frail (34).The ageing process can cause the urethral muscles to lose pressure and pelvic floor muscles to weaken (46). This may lead to decreased storage capacity of the bladder, increased residual urine, and involuntary bladder contraction with age (47). Another important observation of the current study was that patients in the frail group were generally older, and age consistently remained an independent predictor of poor prognosis in multivariate analysis before PSM, which is also consistent with the numerous previous studies that chronologic age has been shown to be a good predictor of adverse post-operative outcomes following surgery in a variety of specialties (48, 49). Similarly, the Charlson comorbidity index (CCI), a validated prospective to comorbidity classification method, has been shown to modify the risk for adverse outcomes in many longitudinal studies (50, 51). A recent analysis of patients undergoing TURP for BPH has demonstrated that men with higher CCI scores were significantly more likely to experience morbidity than men who scored low (52). Indeed, we also observed a higher rate of comorbidities in frail patients, and this might be found to be an independent predictor for poor LUTS improvement in further multivariate analysis. Meanwhile, as frailty discriminant score is significantly associated with comorbidity (e.g., cardiovascular disease, diabetes mellitus, etc.), these observations confirmed that LUTS is also a sign of frailty in the elderly population.

The age and CCI have conventionally been the mainstays for the prediction of adverse events during all aspects for patients undergoing urological procedures. However, these prognostic parameters are only single manifestations of urological disease and do not take into account factors of disease progression. Therefore, we believed that frailty, as an extensively synthetic and integrated physiological system marker, might be a better predictive factor. Our results confirmed that frailty was a stronger predictor of poor postoperative LUTS improvement in BPH patients when performed PSM in our large scale cohort.

Despite its novelty, the current study has several limitations. First, the retrospective nature of the study limited the population size and duration of follow-up. In addition, this was a single-center study. Due to hospital and surgeon-related characteristics, there may be some unobserved confounders not presented in the propensity matching, such as specific internal standards and professional training quality of surgeons, which could influence the outcome. Lastly, using a binary definition of frailty did not allow evaluating the effect of varying degrees of frailty. In light of these regards, a multi-center study on a larger scale should be conducted in great detail to better assess the significance of frailty among these patients. Despite these shortcomings, the present study was instrumental in validating frailty as a predictor of postoperative outcomes after TURP procedures and provides a foundation for future studies.

## Conclusion

To the best of our knowledge, this is the first study to investigate the effect of frailty on postoperative LUTS improvement in patients with BPH undergoing TURP. Our data demonstrates that frail individuals are at higher risk of adverse postoperative LUTS and HRQOL outcomes, and frailty is a strong predictor of poor outcome after TURP. Thus, the frailty has the potential to provide substantial medical risk stratification value and should be assessed carefully perioperatively. Further studies are warranted to consolidate our results.

## Data availability statement

The original contributions presented in the study are included in the article/supplementary material, further inquiries can be directed to the corresponding author.

## Ethics statement

The studies involving human participants were reviewed and approved by Ethics Committee of Tongji Hospital. The patients/participants provided their written informed consent to participate in this study. Written informed consent was obtained from the individual(s) for the publication of any potentially identifiable images or data included in this article.

## Author contributions

XR contributed to preparing and conducting this research. YY and JW made contributions to the acquisition of patient information. ZW carried out the statistical calculation of this study. ZW, JW, XL, YT, and ZG reviewed the manuscript and were involved in its critical revision before submission. XZ designed the research. All authors read and approved the final manuscript.

## Funding

This study was supported by the grants from the National Natural Science Foundation of China (NSFC #82070715), the Fundamental Research Funds for the Central Universities (#YCJJ202201017), and the Natural Science Foundation of Hubei Province (#2021CFB419).

## Conflict of interest

The authors declare that the research was conducted in the absence of any commercial or financial relationships that could be construed as a potential conflict of interest.

## Publisher’s note

All claims expressed in this article are solely those of the authors and do not necessarily represent those of their affiliated organizations, or those of the publisher, the editors and the reviewers. Any product that may be evaluated in this article, or claim that may be made by its manufacturer, is not guaranteed or endorsed by the publisher.
